# Rapid and Efficient Extraction of Cell-Free DNA Using Homobifunctional Crosslinkers

**DOI:** 10.3390/biomedicines10081883

**Published:** 2022-08-04

**Authors:** HyeonAh Seong, Junsoo Park, Minju Bae, Sehyun Shin

**Affiliations:** 1 School of Mechanical engineering, Korea University, Seoul 02841, Korea; 2 Department of Micro-Nano Engineering, Korea University, Seoul 02841, Korea; 3 Engineering Research Center for Biofluid Biopsy, Seoul 02841, Korea

**Keywords:** cfDNA, extraction, homobifunctional, crosslinker, magnetic beads

## Abstract

Since its discovery in circulating blood seven decades ago, cell-free DNA (cfDNA) has become a highly focused subject in cancer management using liquid biopsy. Despite its clinical utility, the extraction of cfDNA from blood has many technical difficulties, including a low efficiency of recovery and long processing times. We introduced a magnetic bead-based cfDNA extraction method using homobifunctional crosslinkers, including dimethyl suberimidate dihydrochloride (DMS). Owing to its bifunctional nature, DMS can bind to DNA through either covalent or electrostatic bonding. By adopting amine-conjugated magnetic beads, DMS–DNA complexes can be rapidly isolated from blood plasma. Using standard washing and eluting processes, we successfully extracted cfDNA from plasma within 10 min. This method yielded a 56% higher extraction efficiency than that of a commercial product (QIAamp kit). Furthermore, the instant binding mechanism of amine coupling between the microbeads and DMS–DNA complexes significantly reduced the processing time. These results highlight the potential of this magnetic bead-based homobifunctional crosslinker platform for extraction of cfDNA from blood plasma.

## 1. Introduction

Since the existence of circulating cell-free DNA (cfDNA) was reported in 1948 [1], it has been buried for a long time; research on this topic has exploded in the last decade owing to elevated concentrations of cfDNA in cancer patients [2,3]. To date, the function and source of cfDNA are not fully known, but apoptosis and necrosis are not the only mechanisms responsible for the presence of such circulating DNA [4]. High concentrations of cfDNA have been found not only in various types of cancer but also in the plasma of patients with many disease states, such as stroke [5], trauma, and myocardial infarction [6], suggesting that cfDNA inhibits inflammation and its release. It appears to be associated with the activation of proinflammatory cytokines [7]. Among cfDNA, circulating tumor DNA (ctDNA) has been further correlated with cancer patients; thus, cfDNA has been intensively investigated for its clinical potential with advances in liquid biopsy technology.

The nature of cfDNA in the blood makes it a challenging analyte to extract. Its first characteristic is the low concentration of cfDNA in plasma, ranging from 1.8 to 44 ng/mL in healthy controls [8] and to 180 ng/mL in cancer patients [9]. Although the average concentration of cfDNA in cancer patients tends to be higher than that in healthy controls, it remains difficult to detect at such low concentrations. Furthermore, ctDNA is frequently less than 0.01% of the total cfDNA [10,11]. Incomplete recovery of cfDNA during the extraction process may result in the loss of targeted ctDNA, which degrades subsequent downstream analyses, including PCR (Polymerase Chain Reaction) and NGS (Next-Generation Sequencing). In addition, cfDNA should be purified as soon as possible because of its short half-life (16–150 min) [12]. Thus, precision liquid biopsy is strongly dependent on high-quality cfDNA extraction.

Currently, cfDNA is extracted using columns or magnetic beads, by utilizing a silica-based salt-bridge binding mechanism [13]. Typical schemes that use silica materials include spin-column silica membranes [14,15] and silica-coated magnetic beads [16,17]. The widely used spin column method is known to provide a high yield and purity of DNA through a repetitive centrifuge workflow. In our previous study, we developed automated cfDNA extraction using microfluidics without the centrifugation process [18,19]. Magnetic beads are microscale sized (0.5–5 µm) and are superparamagnetic. When placed in a magnetic field, they can become magnetized and separate from other. Recently, owing to the rapid development of liquid handling system automation, the manual processing of magnetic silica beads for cfDNA extraction has been automated [20]. However, the inclusion of magnetic beads in the sample solution can result in serious problems, including interference with NGS signals. All these schemes use highly chaotropic salt conditions for binding and low salt concentrations for elution.

On the other hand, there have been studies utilizing non-chaotropic reagents, such as homobifunctional imidoesters (HIs), dimethyl adipimidate (DMA), dimethyl pimelimidate (DMP), dimethyl 3,3′-dithiobispropionimidate (DTBP), and dimethyl suberimidate (DMS), to avoid the use of hazardous chemicals in chaotropic assays [20,21]. HI materials containing two reactive amine groups can bind to nucleic acids (DNA and RNA) via covalent bonding and electrostatic coupling [21,22]. For instance, premixing DTBP with blood plasma results in strong binding between the amine groups of DTBP and cfDNA. The premixed sample is passed through a microchannel, the surface of which is modified with 3-aminopropyl diethoxymethylsilane (APDMS) to bind to the amine groups of DTBP. The other amine group of DTBP then binds to the amine-conjugated surface of the microchannel while flowing.

In this study, we investigated the cfDNA extraction efficiency after applying HI materials to mobile magnetic beads that can freely move and capture cfDNA in a sample solution. Among the HI materials (DMS, DMA, DMP, and DTBP), DMA was carefully selected considering the cfDNA extraction performance, cost, and other factors. The cfDNA extraction performance of the proposed method was compared with that of a commercial spin-column method (QIAamp kit). Furthermore, the optimal concentrations of DMS in plasma and the binding time were examined. This simple, universal, and highly efficient cfDNA extraction method can potentially contribute significantly to studies on liquid biopsy for basic research, as well as clinical diagnostics and treatment monitoring.

## 2. Materials and Methods

### 2.1. Materials

Plasma samples were purchased from Zen-Bio, Inc. (Research Triangle, NC, USA). Unless otherwise specified, a plasma volume of 1 mL was used for downstream analysis. DTBP, DMA, DMS, and DMP used for cfDNA extraction, as well as all other chemicals, were purchased from Sigma-Aldrich (St. Louis, MO, USA). Cell-free DNA ScreenTape Analysis and the 4200 TapeStation System used for cfDNA analysis were purchased from Agilent Technologies (Santa Clara, CA, USA). The DeNovix dsDNA Ultra High Sensitivity Assay Evaluation Kit and DS-11 FX+ spectrophotometer used for DNA analysis were purchased from DeNovix (Wilmington, NC, USA). The QIAamp Circulating Nucleic Acid Kit used for cfDNA extraction was purchased from QIAGEN (Dusseldorf, Germany). The AccuPower 2X GreenStar qPCR Master Mix, PCR Primer, lambda DNA, and the AccuPrep PCR/Gel Purification Kit were purchased from Bioneer (Daejeon, Korea). The CFX96 Touch Real-Time PCR Detection System used for real-time PCR was purchased from Bio-Rad Laboratories (Hercules, CA, USA). The PCR tubes and caps used for real-time PCR were purchased from Thermo Fisher Scientific (Waltham, MA, USA). Experimental equipment, such as pipettes, was purchased from Eppendorf (Hamburg, Germany).

### 2.2. Operating Principles of cfDNA Extraction

**Figure 1:**
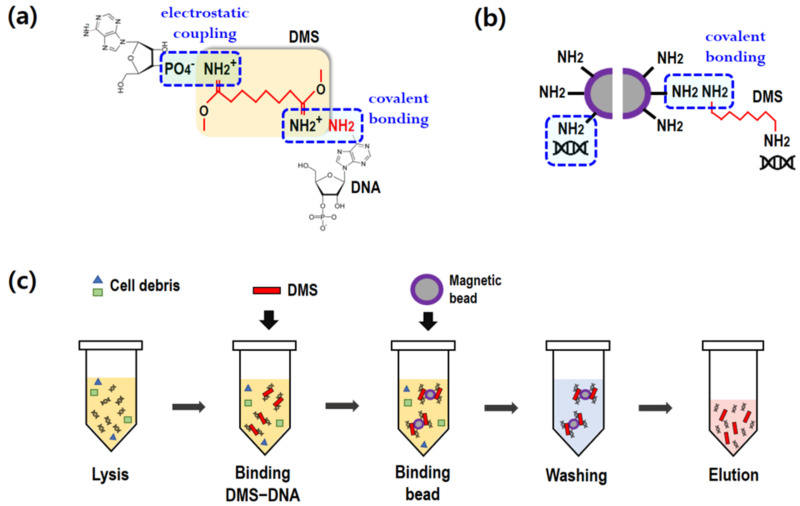
Schematic for cfDNA extraction using a homobifunctional imidoester crosslinker (DMS) and magnetic beads. (**a**) Binding schemes between DMS and DNA. (**b**) Binding between amine-coated magnetic beads and DMS–DNA complexes. (**c**) Standard protocol for cfDNA extraction using the present method.

To extract cfDNA from blood plasma, a two-step process was introduced in the present study: (1) premixing of DMS and blood plasma, and (2) binding of DMS and magnetic beads. As illustrated in Figure 1a,b, DMS serves as a crosslinker between the DNA and magnetic beads. It is notable that the imidoester groups of DMS, containing amine groups, covalently bind to the amine groups of nucleic acids [20]. In addition, the positively charged amine groups of DMS binds to the negatively charged nucleic acids (electrostatic coupling). DMS is a homobifunctional crosslinker with two reactive amine groups. The unbound amine group can bind to the amine-conjugated magnetic beads, as shown in Figure 1b.

Figure 1c depicts the standard protocol for cfDNA extraction using a homobifunctional crosslinker. Generally, cfDNA refers to all non-encapsulated DNA in blood plasma. Lysis buffer containing proteinase K was first mixed with 1 mL of plasma in a 15 mL conical tube and was incubated at 60 °C for 30 min. For the binding process, the DMS solution was added to the plasma. After vortexing for 10 s, DMS immediately bound to DNA. Subsequently, amine-coated beads were added into the plasma to attach to the DMS–DNA complexes. The binding process was conducted at room temperature (RT) using a rocking mixer at 50 rpm. Upon gentle mixing, the magnetic beads and DMS–DNA complexes were effectively bound. After the binding step, a magnet was placed near the tube and the beads were collected within 1 min. The supernatant was then carefully removed using a pipette.

In the washing step, the magnetic beads were washed twice with PBS (2 mL) at pH 7.4 to remove impurities on the complexes, such as cell debris. After carefully removing the supernatant with a magnet, the DMS–DNA complex-bound beads were separated and collected using 0.01 M sodium bicarbonate (100 μL), which was adjusted to pH 10.3. To completely break the crosslinking, the elution buffer was vortexed for 2 min and incubated at RT for 3 min. A magnet was then placed near the tube for 1 min to collect the magnetic beads. The supernatant containing pure cfDNA was finally collected using a pipette.

### 2.3. cfDNA Extraction Using Spin Column

Spin-column-based cfDNA extraction was performed according to the recommended protocol. The reagents in the QIAamp Circulating Nucleic Acid Kit (55114; QIAamp) were used to isolate cfDNA. Lysate buffer (ACL) containing 1 μg of carrier RNA was prepared prior to the experiment. The volume of human plasma varied from 500 μL to 1 mL. Proteinase K solution at one-tenth of the required plasma volume, required human plasma, and ACL at four-fifths of the required plasma volume were sequentially added to a 15 mL conical tube. The mixture was mixed homogeneously by vortexing for 30 s and incubated at 60 °C for 30 min. Binding buffer (ACB) at nine-fifths of the required plasma volume was then added to the mixture. After vortexing for 30 s, the final mixture was incubated on ice for 5 min. The volume of the final mixture varied from 1.85 to 3.70 mL. A spin column with a provided extender was mounted on a QIAvac 24 Plus manifold (QIAGEN), which was connected to a vacuum pump. In the cfDNA binding step, the final mixture was added to the spin column and the vacuum pump was turned on until the final mixture completely passed through the silica membrane. The extender was removed after cfDNA binding. In the washing step, 600 μL of washing buffer 1 (ACW1), 750 μL of washing buffer 2 (ACW2), and 750 μL of 99% ethanol (washing buffer 3) were sequentially passed through the silica membrane. In the drying step, the spin column was centrifuged at 12,000× *g* for 3 min in a 2 mL collection tube, and then transferred to a fresh 1.5 mL elution tube. Finally, in the elution step, 50 μL elution buffer was carefully applied to the center of the spin column and centrifuged at 12,000× *g* for 1 min.

### 2.4. Standard cfDNA Sample Preparation

A cfDNA standard sample was obtained by spiking a 180 bp PCR product from lambda DNA in human plasma collected from healthy controls. The primer set (forward primer: 5-CAGCGATGGATTTTATTCTGG-3; reverse primer: 5-CGTTATCCGTATCCTGAGC-3) was synthesized by Bioneer. Amplification was performed under the following conditions: polymerase activation at 95 °C for 1 min; 40 cycles of denaturation at 95 °C for 10 s; 40 cycles of annealing and extension at 60 °C for 30 s. The PCR product was purified using an AccuPrep PCR/Gel Purification Kit (Bioneer). Purified fragmented DNA from lambda DNA was spiked into 900 μL of healthy human plasma.

### 2.5. Analysis of Isolated cfDNA

#### 2.5.1. Purity, Quantity, and Quality of Extracted cfDNA

The purity of cfDNA was determined by measuring the ratio of the absorbance of 1 µL of the final eluent at 260 and 280 nm in a DS-11 FX+ spectrophotometer (DeNovix). Elution buffer was used as the negative control to blank the spectrophotometer and remove background noise. The DNA concentration was measured using a spectrometer with the DeNovix dsDNA Ultra High Sensitivity Assay Evaluation Kit (DeNovix). The DNA concentration was determined following the recommended protocol. After mixing the sample and reagent from the kit, the absorbance was measured after incubation for 5 min at RT (25 °C). The DNA concentration of the spiked samples was measured with a spectrometer, using the Quant-iT PicoGreen dsDNA Assay Kit (Thermo Fisher Scientific). The fluorescence intensity of PicoGreen was measured to determine the DNA recovery rate of the final eluent. The recovery rate was calculated by standardizing with a known amount of spiked lambda DNA fragments by subtracting the inherent cfDNA amount in the donor. To check the quality of the extracted DNA, we used a TapeStation 4200 automated instrument for microelectrophoresis (Agilent) with Cell-free DNA Screen Tape (Agilent).

#### 2.5.2. Amplification of Extracted DNA

DNA amplification was conducted while targeting *RPLP0* and *GAPDH* as the reference housekeeping genes (Bioneer). DNA extraction was performed using a CFX96 Touch Real-Time PCR Detection System (Bio-Rad), as follows: 1 cycle at 95 °C for 1 min and 95 °C for 30 s, followed by 40 cycles at 60 °C for 30 s. The sample (20 µL) was placed in a transparent 0.1 mL 8-tube strip (Thermo Fisher Scientific) and sealed with an 8-strip cap (Applied Biosystems, Waltham, MA, USA). Data were analyzed using Bio-Rad CFX Maestro software, and the cycle threshold (Ct) was set to 400.

## 3. Results

### 3.1. Selection of HI Crosslinkers

**Figure 2:**
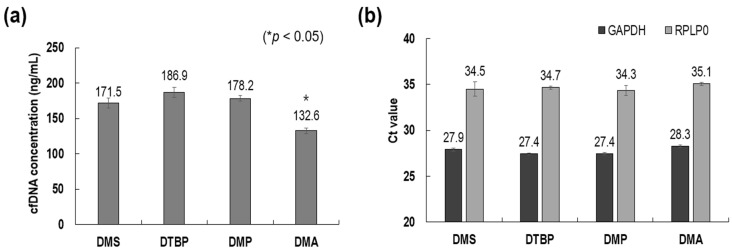
Comparison of cfDNA extraction with different homobifunctional imidoester crosslinkers (DMS, DTBP, DMP, and DMA). (**a**) cfDNA concentrations. (**b**) Threshold cycles for two house-keeping genes (*GAPDH* and *RPLP*0).

To evaluate the extraction performance of cfDNA using HI crosslinkers in detail, it was necessary to select one of the HI materials, namely, DMS, DMA, DMP, and DTBP. With a fixed concentration (30 mg/mL) and the same protocol, the four materials were compared in terms of cfDNA extraction performance using a TapeStation and PCR. As shown in Figure 2, DTBP showed the highest yield of cfDNA extraction, although the yields were not significantly different except in terms of DMA. In the PCR analysis with two housekeeping genes (*GAPDH* and *RPLP*0), all four materials showed nearly the same Ct values within a standard deviation of 0.5. Through the technical performance analyses of cfDNA extraction, we confirmed that there was no significant difference among the four HI crosslinkers. Therefore, DMS was selected considering only the material cost, and all subsequent results presented are those for DMS, unless otherwise specified.

### 3.2. Comparison of Recovery Yield, Size, and Purity of Extracted cfDNA

**Figure 3:**
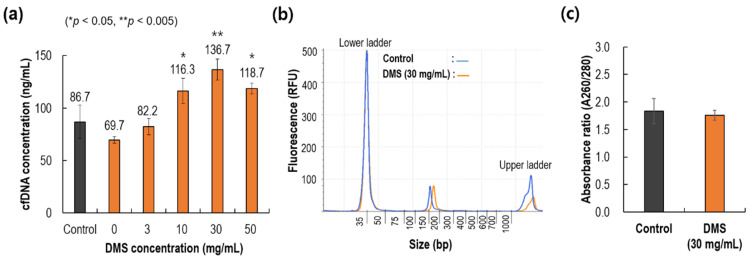
Comparison of cfDNA extraction performance with varying DMS concentrations. (**a**) Effect of DMS concentration (0, 3, 10, 30, and 50 mg/mL) on cfDNA extraction yields compared to that of control (QIAamp). (**b**) Size analysis of cfDNA extracted from control and present sample (30 mg/mL). (**c**) Absorbance ratios for control and present sample (30 mg/mL).

As shown in Figure 1c, the current process of cfDNA extraction from plasma is identical to the general protocol for cfDNA extraction using magnetic beads. After lysis, DMS was added to the samples, followed by amine magnetic beads. In general, it is known that the binding between DMS and cfDNA is very fast. As a result of testing in this study (Figure S1), it was evaluated that all binding was completed within 1 min. Firstly, we examined the effect of DMS concentration on cfDNA extraction from the plasma samples. For comparison, we used a commercial kit (QIAamp, QIAGEN) as the control. As illustrated in Figure 3a, the extracted cfDNA concentrations, which were measured with a TapeStation, were compared for five different concentrations of DMS and the control. The DMS concentration of 30 mg/mL showed the highest yield (136.7 ng/mL) of cfDNA extraction from plasma, 56% higher than that of the control (86.7 ng/mL). The result for the highest concentration of DMS (50 mg/mL) was slightly inferior to that for 30 mg/mL, and such results were frequently observed [23].

As the DMS concentration increased, the recovered cfDNA showed a maximum at 30 mg/mL rather than 50 mg/mL, which could be explained by the salting-out effect [24]. Similar results were observed in a previous study to isolate exosomes from plasma by adding NaCl [25]. In general terms, salting out is the phenomenon observed when the solubility of a nonelectrolyte compound in water decreases with an increase in the concentration of a salt. This salting-out is generally explained by a combination of electrostatic repulsion and an enhancement of the hydrophobic effect [26]. Similarly, as the concentration of DMS increased, DMS–DNA binding decreased and DNA self-aggregation increased due to the salting-out effect. Therefore, the original purpose of DMS as a crosslinking agent was rather weakened at high DMS concentrations.

As shown in Figure 3b, the size of the extracted DNA was examined using a TapeStation analyzer. It is notable that cfDNA size ranges 120–220 bp, with an average of 181 bp. Although there was a slight difference in the two peaks of the control and the present DMS method, the results confirmed that the DNA extracted with the two methods was cfDNA. The control is a silica-based spin column method, whereas the present method is a DMS-based magnetic bead method. In addition, as shown in Figure 3c, the absorbance ratios of the two methods were around 1.8, which confirmed the purity of the extracted samples.

### 3.3. Effects of Binding Time and Bead Concentration on cfDNA Extraction

**Figure 4:**
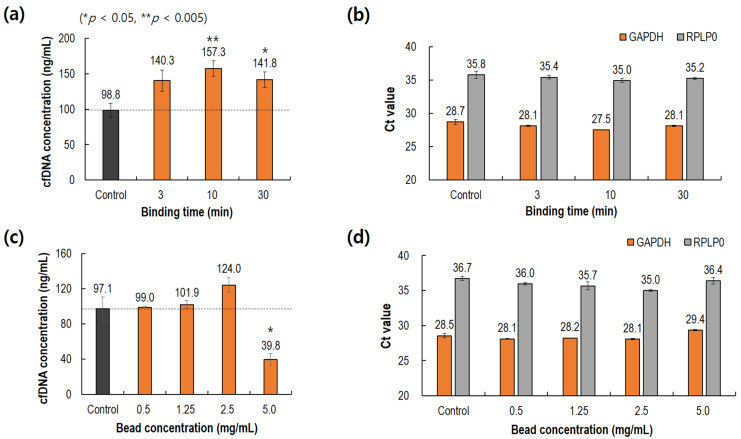
Comparison of cfDNA extraction with varying binding time and bead concentration. (**a**) Effect of binding time on cfDNA recovery. (**b**) Effect of binding time on cycle threshold of PCR. (**c**) Effect of bead concentration on cfDNA recovery. (**d**) Effect of bead concentration on cycle threshold of PCR. Control: QIAamp (Qiagen).

As illustrated in Figure 1c, there is a step for binding between the DMS–DNA complexes and amine magnetic beads, which is amine–amine covalent bonding. This binding step was required to optimize the binding time and bead concentration. Unlike the binding between DNA and DMS, amine–amine covalent bonding requires a relatively long binding time. cfDNA recovery was examined by varying the binding time (3, 10, and 30 min). As shown in Figure 4a, 10 min of binding time showed the highest recovery yield, although the difference was not statistically significant. All the DMS results showed a higher recovery yield than that of the control. A similar result was found in the PCR analysis for amplifying the two genes. The 10 min binding time showed the fastest PCR amplification without statistical significance.

Figure 4c,d depict the effect of bead concentration (0.5, 1.25, 2.5, and 5.0 mg/mL) on cfDNA extraction using the DMS crosslinker. The 2.5 mg/mL bead concentration showed the best recovery yield. In addition, the PCR results shown in Figure 4d were similar to the cfDNA recovery results. In Figure 4c, as the bead concentration increased; the recovered cfDNA showed a maximum at 2.5 mg/mL rather than 5.0 mg/mL. These results could be also explained by the salting-out effect [24]. Even though there was successful DNA–DMS binding, high concentrations of magnetic beads might cause another salting-out effect, which led to a reduction in the extraction of DMS–DNA complexes through magnetic beads.

### 3.4. Performance Comparison of Spiked DNA in Plasma

**Figure 5:**
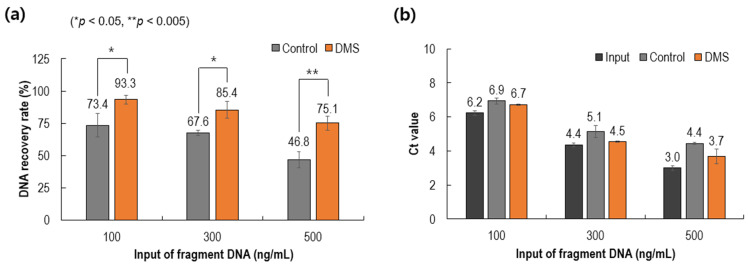
Performance analysis of cfDNA extraction. (**a**) DNA recovery rates according to input of fragment DNA. (**b**) Threshold cycles for the spiked DNA according to input of fragment DNA. Control: QIAamp (Qiagen).

As depicted in Figure 5, we prepared standard samples by spiking a 180 bp PCR product from lambda DNA in human plasma collected from healthy controls. By varying the concentration (100, 300, and 500 ng/mL) of the spiking lambda DNA, the recovery yield of target DNA for a commercial product (QIAamp) and the present product was compared. At 100 ng/mL of input DNA spiking, the yield of QIAamp was 73.4%, whereas the present yield was 93.3% (27.1% higher than QIAamp). With an increase in the input concentration of spiked DNA, the recovery yields of both methods tended to decrease. Additionally, PCR analyses of the spiked DNA in the two methods were compared, with reference to the input sample. The DMS method showed smaller Ct values than the QIAamp method.

## 4. Discussion

The various advantages of liquid biopsy have generated research and clinical interest over the past decade. Among them, the ease of blood sampling may enable longitudinal monitoring of cancer and an analysis of the response to treatment. However, liquid biopsy suffers from the low concentration of cfDNA in the blood and the low frequency of ctDNA among cfDNA. Therefore, low recovery yields of cfDNA from a sample may miss the low concentration of the target ctDNA in the sample preparation process. It is notable that since the extraction process is preceded by downstream detection or sequencing, the extraction yield may strongly affect the precision of the downstream processes. Table 1 summarizes the recovery rates for various technologies and methods proposed in a recent decade.

Among them, the spin-column method, as used in the QIAamp circulating nucleic acid kit (QIAGEN), has been widely used in laboratories because it is easily accessible through equipment commonly equipped in laboratories such as centrifuges. However, the recovery rates of QIAamp ranged from 60–80% [23], which did not meet the needs of studies targeting low-concentration cfDNA. On the other hand, the microfluidic methods [19,20,27] succeeded in improving the extraction time, but there was no particular improvement in extraction efficiency. In fact, the extraction efficiency is strongly dependent on the materials for DNA extraction. In terms of such extraction efficiency, the present study demonstrated remarkable results yielding 93% and hence, it is necessary to delineate the cause of the enhanced cfDNA extraction rate of the DMS method.

Crosslinkers can be classified as homobifunctional and heterobifunctional. The former have two identical reactive functional groups at either end, making them ambidextrous. Such reagents can link the target material to another by covalently reacting with a common group of two molecules. Reactive ends are chosen for their particular chemoselective properties, often targeting the primary amines and sulfhydryl groups of proteins. The present study adopted an amine group as the reactive end, which is known for HI crosslinkers, including DTBP, DMA, DMP, and DMS. In other words, DMS, which contains two identical amine groups, ties DNA to amine magnetic beads using the two functional reactive amine groups at both its ends. Furthermore, owing to the positively charged nature of the amine group in DMS, DNA can bind to DMS via electrostatic coupling, as illustrated in Figure 1. If DNA can bind to both ends of DMS via either covalent or electrostatic binding, the amine group contained in DNA can bind to the amine surface of the magnetic beads. As shown in Figure 1b, there is direct binding between DNA and magnetic beads, which was shown to contribute approximately 50% of the maximum DNA recovery (0 mg/mL of DMS in Figure 3a). In other words, direct bonding between amine beads and DNA should be considered when designing DNA capture. However, its recovery was still lower than that of the control, and this increase in cfDNA recovery yield using DMS could be mainly due to the features of HIs: (1) two reactive functional groups at both ends and (2) the reactive functional amine group.

Another feature of the proposed method is the shortened processing time. The conventional spin-column method requires a lengthy process (1–2 h) and skilled manual operations including repeated pipetting and centrifugation. In fact, repetitive pipetting and long experimental times run the risk of contaminating the sample. Meanwhile, the present method could complete the same process within 20 min, including binding, washing, and elution. The longest step was the binding between the DMS–DNA complexes and magnetic beads (10 min). The fast process is mainly due to the nature of HI crosslinkers, whose binding is nearly instant. Furthermore, there have been rapid developments in automation techniques for liquid handling systems. The previously developed manual operating system with magnetic beads rapidly migrated to an automated system using disposable pipette tips and an automated liquid handler. The ease of operation of an automated system may accelerate the use of magnetic beads in sample preparation. However, it is notable that any inclusion of magnetic beads in the final samples can result in signal interference in NGS analyses; therefore, the magnetic beads should be completely excluded in the final eluted samples.

This study had some limitations, which necessitate additional research. Firstly, we only investigated the potential of homobifunctional crosslinkers with amine groups; it is necessary to investigate homobifunctional crosslinkers with other reactive groups. However, the target materials, including DNA and proteins, and their functional groups should be considered. The functional groups of DNA are amine (-NH_2_), amide (-CONH-), hydroxyl (-OH), glycoside linkage (O-C-N-), and phosphodiester groups. Considering these functional groups, various crosslinking reagents can be synthesized, and the potential for cfDNA extraction must be examined. Secondly, the effect of magnetic bead size on the recovery yield of cfDNA extraction should be examined. The size of the present magnetic beads was 1 μm, which is known as the minimum size for the efficient collection of microbeads with a magnet. However, owing to the development of encapsulated magnetic material in a bead, nano-sized magnetic beads can be rapidly collected by a magnet within a few seconds. Hence, it is worthwhile to examine the potential of smaller magnetic beads rather than microscale beads.

## 5. Conclusions

The present study demonstrated the potential of HI crosslinkers to extract cfDNA from plasma using magnetic beads. Compared to the silica-based spin-column method, the present method showed a significantly superior yield of cfDNA, which was mainly due to the nature of the HI crosslinkers, which contained twin amine functional groups at both ends. Since the HI crosslinkers were used to provide covalent bonding and electrostatic coupling via amine functional groups, cfDNA efficiently bound to the crosslinkers, which were further efficiently bound to the magnetic amine beads. In addition, the use of the HI crosslinker shortened the entire extraction process time to within 20 min because covalent bonding is a nearly instant reaction. The current method is expected to be applied to basic research and clinical applications that require cfDNA extraction. As the current method exhibits a high performance for cfDNA extraction, it might play a significant role in the advancement of cfDNA-based basic research, including research on biomarker discovery, as well as clinical applications, such as molecular diagnostic methods and treatment monitoring strategies. Additionally, the present method is expected to further facilitate the application of a final sample-to-answer system in liquid biopsy to address unmet clinical needs in cancer management.

## Figures and Tables

**Table 1:** Summary of cfDNA extraction.

Category	Material	Description	Recovery Rate	Ref.	Year
Magnetic bead	Amine-modified bead	Homobifunctional Imidoester	93%	Present study	
Membrane	Silica	PIBEX	80%	[19]	2020
Membrane	Silica	QIAamp circulating nucleic acid kit (QIAGEN)	80%	[23]	2018
Magnetic bead	Cellulose	Maxwell RSC ccfDNA plasma kit (Promega)	60%		
Magnetic bead	Unknown	MagMax cell-free DNA isolation kit (Applied Biosystems)	50%		
Bead	Silica	Rotating disc	75%	[27]	2018
Micro-channel	Amine-modified form	Adding DTBP	-	[20]	2018

## Data Availability

The authors confirm that the data supporting the findings of this study are available within the article [and/or] its Supplementary Materials.

## Supplementary Materials

The following are available online at https://www.mdpi.com/article/10.3390/biomedicines10081883/s1, Figure S1: Comparison of cfDNA extraction with varying DMS-DNA binding time. Figure S2: Comparison of cfDNA extraction performance with two different bead sizes.
