# Supplementary material for: Rapid and Efficient Extraction of Cell-Free DNA Using Homobifunctional Crosslinkers
Source: Biomedicines. 2022 Aug 4;10(8):1883. doi: 10.3390/biomedicines10081883 (PMC9405790; doi:10.3390/biomedicines10081883)
Supplement: Supplementary file 1 [file biomedicines-10-01883-s001.zip › biomedicines-1822831-supplementary.pdf]

biomedicines

Article

Rapid and Efficient Extraction of Cell-free DNA Using Homobifunctional Crosslinkers

HyeonAh Seong [1+], Junsoo Park [2+], Minju Bae [1] and Sehyun Shin [1,2,3*]

[1] School of Mechanical engineering, Korea University, Seoul, Korea
[2] Department of Micro-Nano engineering, Korea University, Seoul, Korea
[3] Engineering Research Center for Biofluid Biopsy, Seoul, Korea
* Correspondence: lexerdshin@korea.ac.kr; Tel.: +82-10-4506-2825

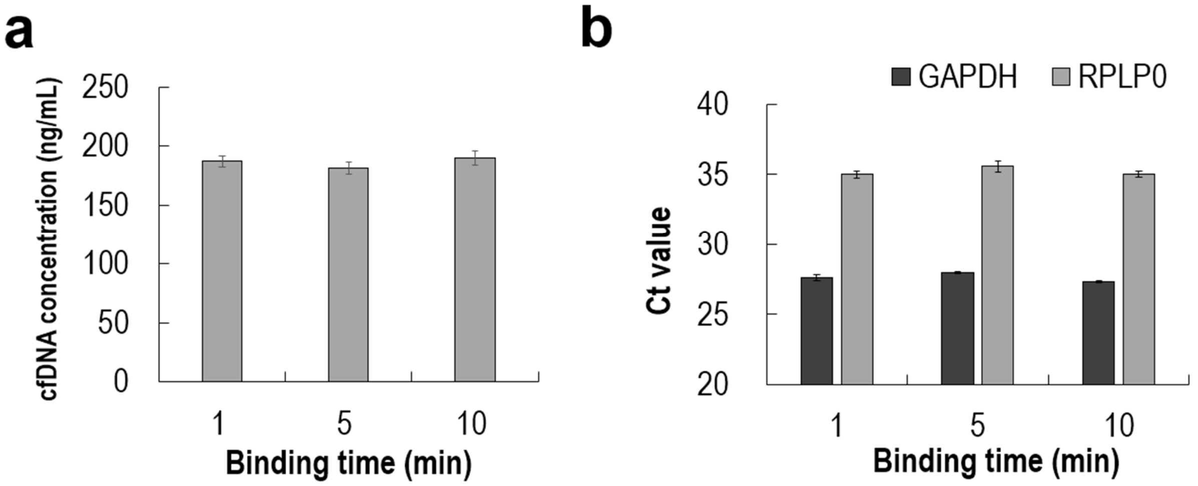

Figure S1 Comparison of cfDNA extraction with varying DMS-DNA binding time. a) Effect of DMS-DNA binding time on cfDNA recovery. b) Effect of DMS-DNA binding time on cycle threshold of PCR.

biomedicines

MDPI

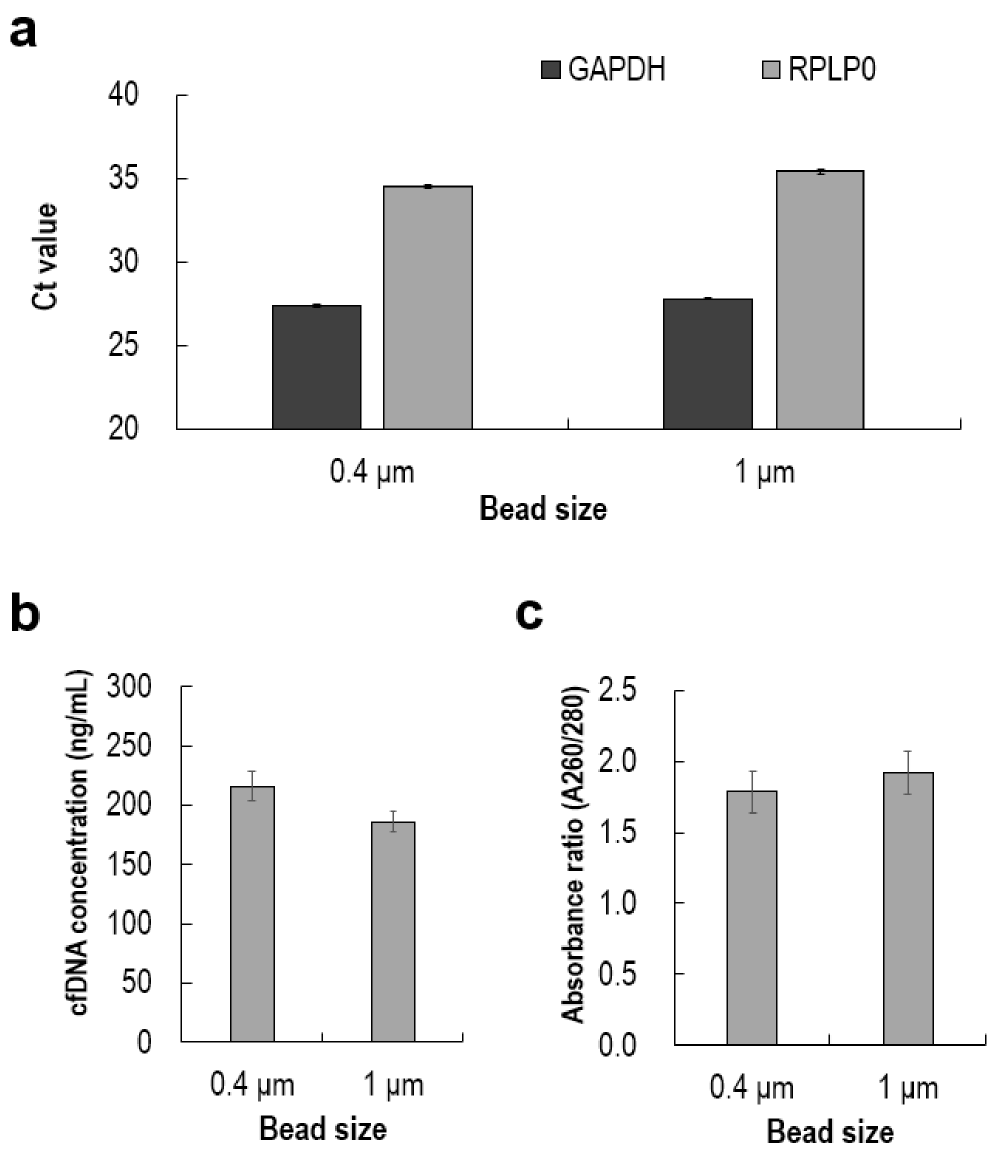

Figure S2 Comparison of cfDNA extraction performance with two different bead sizes. a) Effect of bead size on cycle threshold of PCR. b) Effect of bead size on cfDNA concentration. c) Effect of bead size on cfDNA purity ratio.
